# Breast cancer risk prediction using machine learning: a systematic review

**DOI:** 10.3389/fonc.2024.1343627

**Published:** 2024-03-20

**Authors:** Sadam Hussain, Mansoor Ali, Usman Naseem, Fahimeh Nezhadmoghadam, Munsif Ali Jatoi, T. Aaron Gulliver, Jose Gerardo Tamez-Peña

**Affiliations:** ^1^ School of Engineering and Sciences, Tecnologico de Monterrey, Monterrey, Mexico; ^2^ Department of Electrical and Computer Engineering, University of Victoria, Victoria, BC, Canada; ^3^ College of Science and Engineering, James Cook University, Cairns, QLD, Australia; ^4^ Institute of Obesity Research, Tecnològico de Monterrey, Monterrey, Mexico; ^5^ Department of Biomedical Engineering, Salim Habib University, Karachi, Pakistan; ^6^ School of Medicine and Health Sciences, Tecnològico de Monterrey, Monterrey, Mexico

**Keywords:** breast cancer, risk prediction, deep learning, digital mammography, clinical factors

## Abstract

**Background:**

Breast cancer is the leading cause of cancer-related fatalities among women worldwide. Conventional screening and risk prediction models primarily rely on demographic and patient clinical history to devise policies and estimate likelihood. However, recent advancements in artificial intelligence (AI) techniques, particularly deep learning (DL), have shown promise in the development of personalized risk models. These models leverage individual patient information obtained from medical imaging and associated reports. In this systematic review, we thoroughly investigated the existing literature on the application of DL to digital mammography, radiomics, genomics, and clinical information for breast cancer risk assessment. We critically analyzed these studies and discussed their findings, highlighting the promising prospects of DL techniques for breast cancer risk prediction. Additionally, we explored ongoing research initiatives and potential future applications of AI-driven approaches to further improve breast cancer risk prediction, thereby facilitating more effective screening and personalized risk management strategies.

**Objective and methods:**

This study presents a comprehensive overview of imaging and non-imaging features used in breast cancer risk prediction using traditional and AI models. The features reviewed in this study included imaging, radiomics, genomics, and clinical features. Furthermore, this survey systematically presented DL methods developed for breast cancer risk prediction, aiming to be useful for both beginners and advanced-level researchers.

**Results:**

A total of 600 articles were identified, 20 of which met the set criteria and were selected. Parallel benchmarking of DL models, along with natural language processing (NLP) applied to imaging and non-imaging features, could allow clinicians and researchers to gain greater awareness as they consider the clinical deployment or development of new models. This review provides a comprehensive guide for understanding the current status of breast cancer risk assessment using AI.

**Conclusion:**

This study offers investigators a different perspective on the use of AI for breast cancer risk prediction, incorporating numerous imaging and non-imaging features.

## 1 Introduction

Breast cancer is the most prevalent cancer in women. In 2020, approximately 2,300,000 new cases were identified worldwide, leading to approximately 688,000 fatalities (1, 2). Projections for 2023 indicate that the United States alone will witness 1,958,310 new cancer cases and 609,820 cancer-related deaths (3). Cancer incidence varies across countries, regions, ethnicities, and lifestyles. Traditional risk prediction models use statistical approaches combined with patient demographic information to predict the risk, recurrence, and survivability of breast cancer (BC). While modest in performance, these techniques often suffer from racial bias (4, 5). Recently, AI has shown promising outcomes in BC risk prediction (6), prognosis (7), recurrence (8, 9), and survival prediction (10), outperforming the traditional models (11). With improved prognoses, patients may have extended survival times (12).

Precise assessment of an individual woman’s risk of breast cancer is essential for customizing screening and preventive measures according to the specific risk levels. Acknowledging the importance of early breast cancer detection and risk categorization, several models, including Gail, BCSC, Rosner–Colditz, and Tyrer–Cuzick, have been developed to predict breast cancer risk (13).

A crucial element of comprehensive breast cancer screening initiatives is evaluating the risk of breast cancer. This assessment helps to identify individuals who could benefit from early and supplementary screening methods, genetic testing, or preventive therapies. It also aids the general population in making informed decisions regarding screening (14–16). Various risk prediction models have been devised to estimate the likelihood of breast cancer occurrence over a specific timeframe in generally healthy women or the probability of having a BRCA1 or BRCA2 mutation (17). These models vary in terms of the specific risk factors considered and their respective weights. Moreover, their performance may differ based on population characteristics since each was developed using specific inclusion criteria. Ongoing research indicates that both traditional risk factors and mammographic images offer complementary information, and AI models based on DL have the potential to enhance existing epidemiological models (18).

In previous reviews within this field, Acciavatti et al. (19) conducted a comprehensive overview of the usage of DL techniques in different imaging modalities, ranging from tomography and mammography to MRI and ultrasound. Their review covered a wide range of risk modeling techniques currently employed in practice. Similarly, investigators (20) have focused on the use of DL techniques, specifically mammography, for breast density assessment and risk analysis. Recently, the authors (21) conducted a narrative review focusing specifically on convolutional neural network (CNN) applications in digital mammography. Furthermore, the authors examined the patterns in scale, structure, risk elements, and medical factors that could potentially affect the effectiveness of CNNs in evaluating the risk of breast cancer. Collectively, these reviews provide valuable insights into the diverse landscape of DL methods and their applications in medical imaging and risk assessment of breast cancer. However, the aforementioned researchers limited their work to imaging or non-imaging modalities and focused on individual imaging modalities.

Therefore, in this systematic review, we comprehensively analyzed both AI-based imaging and non-imaging risk prediction models that contribute to breast cancer risk prediction. We also discuss how state-of-the-art (SOTA) NLP-based techniques can be applied to breast cancer risk prediction.

**Figure 1:**
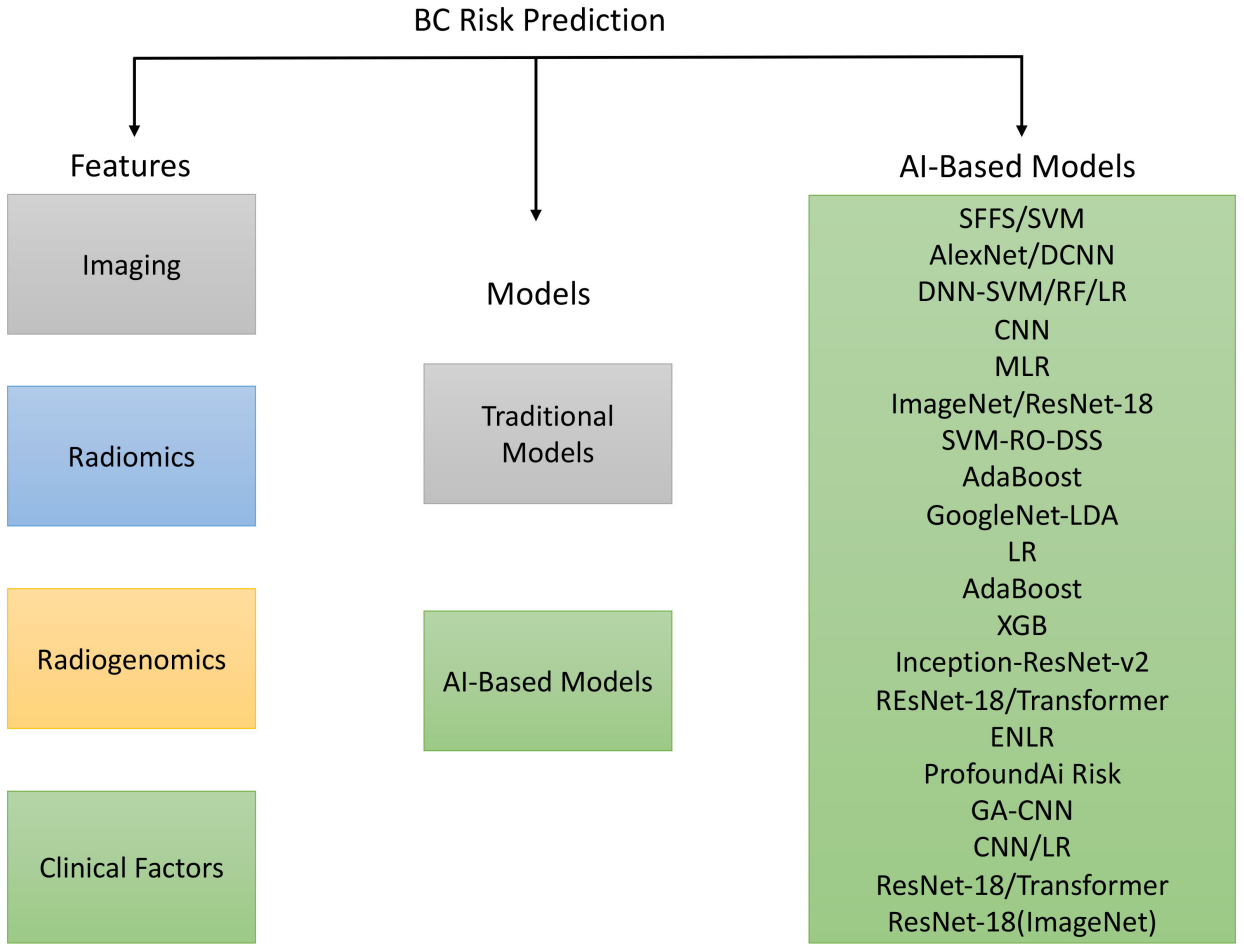
Overview of the features, traditional as well as AI-based models for breast cancer prediction.

The rest of the paper is outlined as follows: *Section 2* describes the methodology for selecting papers; *Section 3* presents the results of imaging and non-imaging features used in risk prediction, conventional and AI-based risk prediction models, and AI in breast cancer risk assessment (see **Figure 1**). A detailed discussion of the previous methods and their limitations is provided in *Section 4*, and the conclusion is presented in *Section 5*.

## 2 Methods

### 2.1 Search strategy

**Figure 2:**
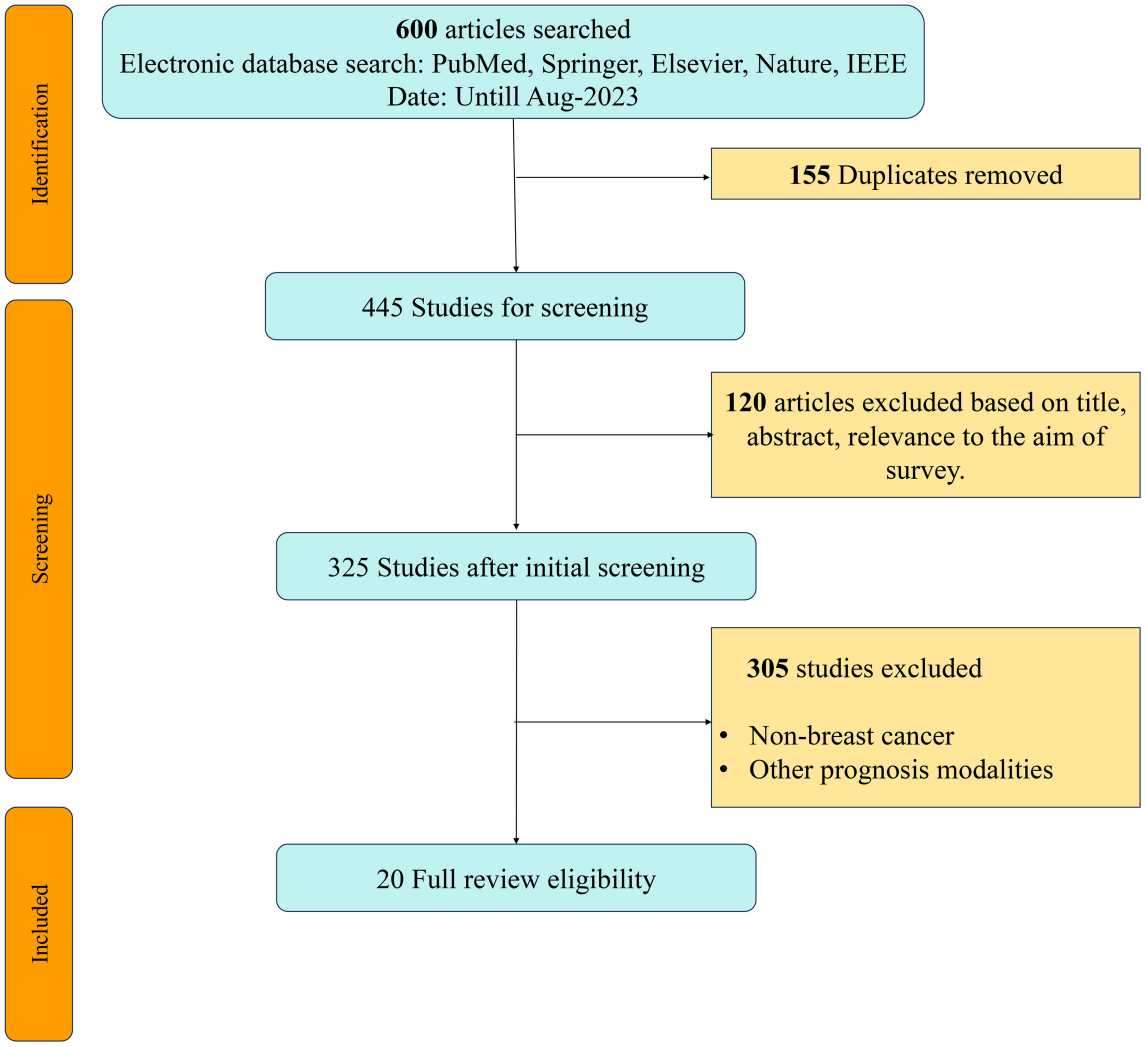
PRISMA Chart showing search methodology.

For this systematic review, articles focusing on AI usage in breast cancer risk prediction were selected from databases such as PubMed, ScienceDirect (Elsevier), Springer, Nature, and IEEE. We included all studies on AI in breast cancer risk prediction up to August 2023, and all selected studies were conducted in English. Our search terms were derived from the key concepts in the review questions, and we followed a detailed plan for the inclusion and exclusion of studies. The keywords for paper selection were: “Deep Learning” AND “Breast Cancer,” “Risk Prediction” AND “Radiological Reports,” “Machine Learning” AND “Risk Assessment,” and “Breast Cancer” AND “Digital Mammography.” These terms were chosen to encompass the scope of this survey. The first, second, and third searches yielded 300, 190, and 110 articles, respectively. The papers were shortlisted based on their titles, abstracts, and text. We reviewed the first 300 articles on machine learning (ML) for breast cancer (BC) risk prediction, focusing on those used for risk prediction. The best methods for each study were summarized and listed. This study followed the PRISMA checklist/flowchart method as highlighted in the PRISMA diagram (see **Figure 2**).

### 2.2 Inclusion criteria

The inclusion criteria for this systematic review were as follows: (1) published peer-review studies; (2) research related to short-term and long-term breast cancer risk prediction; (3) research investigating the use of ML algorithms in breast cancer risk assessment; (4) research related to SOTA DL architectures for risk prediction purposes; (5) algorithms that used internal or external validation for reporting the results; and (6) studies published only in English.

### 2.3 Exclusion criteria

The exclusion criteria were as follows: (1) studies that used ML or DL for prognostic purposes; (2) studies related to BC recurrence or survival prediction; (3) literature reviews; (4) studies not related to human (women) populations; (5) case studies; and (6) editorial reports.

## 3 Results

### 3.1 Imaging and non-imaging features used in risk prediction

In this section, we describe various imaging and non-imaging features utilized for breast cancer risk prediction. These include features extracted from different imaging modalities, as well as non-imaging features, such as radiomics, genomics, and clinical features.

#### 3.1.1 Imaging features in risk prediction

Risk assessment methods for breast cancer incorporate various imaging features and clinical factors to estimate individualized cancer risk and recurrence. The key imaging features include the following:

Mammographic Density: This measures the proportion of fibroglandular tissue on mammograms, indicating saturation of the display signal (22, 23). Higher mammographic density is associated with increased breast cancer risk and reduced mammography recall effectiveness (13, 24).

Mammographic Texture: This refers to the variation in pixel intensity in mammograms, which assesses the spatial arrangement of the breast tissue. Techniques such as fractal dimension, gray-level co-occurrence matrix, and local binary patterns are used to measure mammographic texture. Combining these features with mammographic density can enhance risk prediction (25, 26).

Mammographic Calcifications: Bright areas on mammograms indicate breast cancer. Specific types, such as pleomorphic or linear-branching calcifications, are associated with a higher risk. The authors also suggested the presence of BRCA1 or BRCA2 mutations (24).

Breast MRI Features: Breast MRI is superior to mammography, especially for women with dense breasts or those at high risk for mutations. It provides detailed information on lesion morphology, enhancement patterns, kinetics, and tissue volume. Furthermore, studies show that MRI features can also be predictive of breast cancer risk (24, 27, 28).

#### 3.1.2 Radiomics features in risk prediction

Radiomics is an economical and noninvasive method that utilizes quantitative image attributes from various imaging modalities such as mammography, ultrasound, MRI, and PET to understand breast cancer traits (29–31). These radiomics features reflect tumor heterogeneity and microenvironment, providing insights into breast cancer risk and outcomes (31, 32). Key radiomics features include the following:

Multi-scale Gaussian Features: These analyze texture resemblances at different scales in mammograms, aiding in enhancement of breast cancer risk prediction (30).

Ultrasound-Based Features: Focusing on echogenicity, shape, and other ultrasound characteristics, these features help differentiate between benign and malignant lesions and predict disease-free survival in invasive breast cancer (31).

MRI-Based Features: These features consider MRI lesion characteristics and breast tissue, assisting in predicting molecular subtypes, recurrence risk, lymph node status, and breast cancer risk (28, 32).

#### 3.1.3 Genomics features in risk prediction

Genomics plays a crucial role in assessing the risk of breast cancer by examining genetic mutations, gene expression, protein activity, and epigenetic changes. Examples of genomic features in breast cancer risk estimation include the following:

Genetic Mutations: Mutations in BRCA1 and BRCA2 significantly increased breast cancer risk (33). Other genes, such as PALB2 and CHEK2, also increase this risk (34). Genomic tests can detect these mutations and estimate their likelihood of carrying them based on family history (34).

Gene Expression: Gene expression levels provide insights into tumor characteristics, such as hormone receptor status, invasiveness, and metastatic potential. Gene expression profiles help to classify breast tumors into subtypes, each with distinct outcomes (35).

Protein Expression: Specific proteins such as EIF4G and X4EBP1-pT70 can predict patient survival by influencing critical pathways (36).

Epigenetic Modifications: Changes in DNA methylation and histones play a role in gene regulation and can predict breast cancer recurrence and survival (37).

#### 3.1.4 Clinical features in risk prediction

Non-imaging risk factors unrelated to breast tissue appearance on mammograms include the following:

Family History: A family history of ovarian and breast cancer, especially among first-degree relatives of a young age, indicates an increased risk. This suggests the inheritance of gene mutations, such as BRCA1 or BRCA2 (13).

Genetic Factors: Pathogenic variants in genes such as BRCA1 and BRCA2, or polygenic risk scores derived from common genetic variants, influence breast cancer development by affecting various cellular pathways (34).

Lifestyle and Reproductive Factors: Factors such as parity, age at menarche, breastfeeding, hormone replacement therapy, menopausal status, body mass index (BMI), alcohol consumption, dietary choices, and physical activity can affect hormone exposure, inflammation, oxidative stress, epigenetic changes, and microbiome composition, all contributing to breast cancer risk (38).

Ongoing research continues to explore additional features and integrate imaging characteristics with other risk factors, such as family history, age, genetic mutations, lifestyle, and reproductive history, for comprehensive and tailored risk assessment (13, 24).

### 3.2 Risk prediction models

#### 3.2.1 Traditional risk prediction models

Numerous breast cancer risk analysis techniques utilize a variety of genetic and non-genetic characteristics to estimate the individualized risk of developing breast cancer in women. Various breast cancer risk assessment models cater to different clinical scenarios and populations. The Breast Cancer Risk Assessment Tool (BCRAT), or modified Gail model, evaluates short-term and long-term breast cancer risk using factors such as race, age, and reproductive history, but has limitations like age and cancer history restrictions (13). The Breast Cancer Surveillance Consortium (BCSC) model incorporates breast density as a risk factor but lacks genetic information (24). The Rosner–Colditz model accounts for factors including age, BMI, and family history for 5-year and lifetime risk estimations (24). The Tyrer–Cuzick model (IBIS) predicts long-term risk with a broader range of factors, but requires more data (24, 39). The Claus model relies solely on family history of lifetime risk (24). The BRCAPRO model combines personal and family histories to assess BRCA mutations and cancer risk (24). The BOADICEA model includes a wide array of factors, such as genetic test results, to evaluate BRCA mutations and cancer risk (24, 39). The Myriad model, specifically for BRCA mutations, does not provide risk estimates for other cancer types (24). Each model is suited to different scenarios and populations but has its own limitations. For instance, the BCRAT is often used to determine chemoprevention eligibility but has age and history restrictions. Meanwhile, the BCSC model is more precise for women with dense breasts but lacks genetic input. The IBIS model is comprehensive but data-intensive, the Claus model is straightforward but overlooks non-genetic factors, and the BRCAPRO and BOADICEA models are beneficial for genetic counseling but may not be accurate for individuals without a strong family history. The Myriad method, while focusing on BRCA mutations, omits estimates of breast and ovarian cancer risk.

#### 3.2.2 AI-based risk prediction models

AI has revolutionalized breast cancer risk analysis by enhancing screening and diagnostic accuracy. It interprets mammographic images to identify risk-related features, such as breast density, mass characteristics, texture, and location. Additionally, AI can incorporate factors such as age, family history, and genetics to provide personalized risk scores, showing significant promise for breast cancer screening (40–42).

Data-driven techniques, notably DL and CNNs, are commonly used in AI. CNNs, a subset of DL, handle image-based tasks such as detection, segmentation, and classification, and are capable of predicting breast cancer risk from mammograms alone or in conjunction with additional factors (43–46).

However, the successful integration of AI into clinical workflows and clear communication between patients and providers is crucial for its proper utilization. Although AI offers exciting prospects for breast cancer prevention and early detection, addressing technical, ethical, and practical challenges through further research is key to its broader clinical adoption.

### 3.3 AI in breast cancer risk assessment

**Figure 3:**
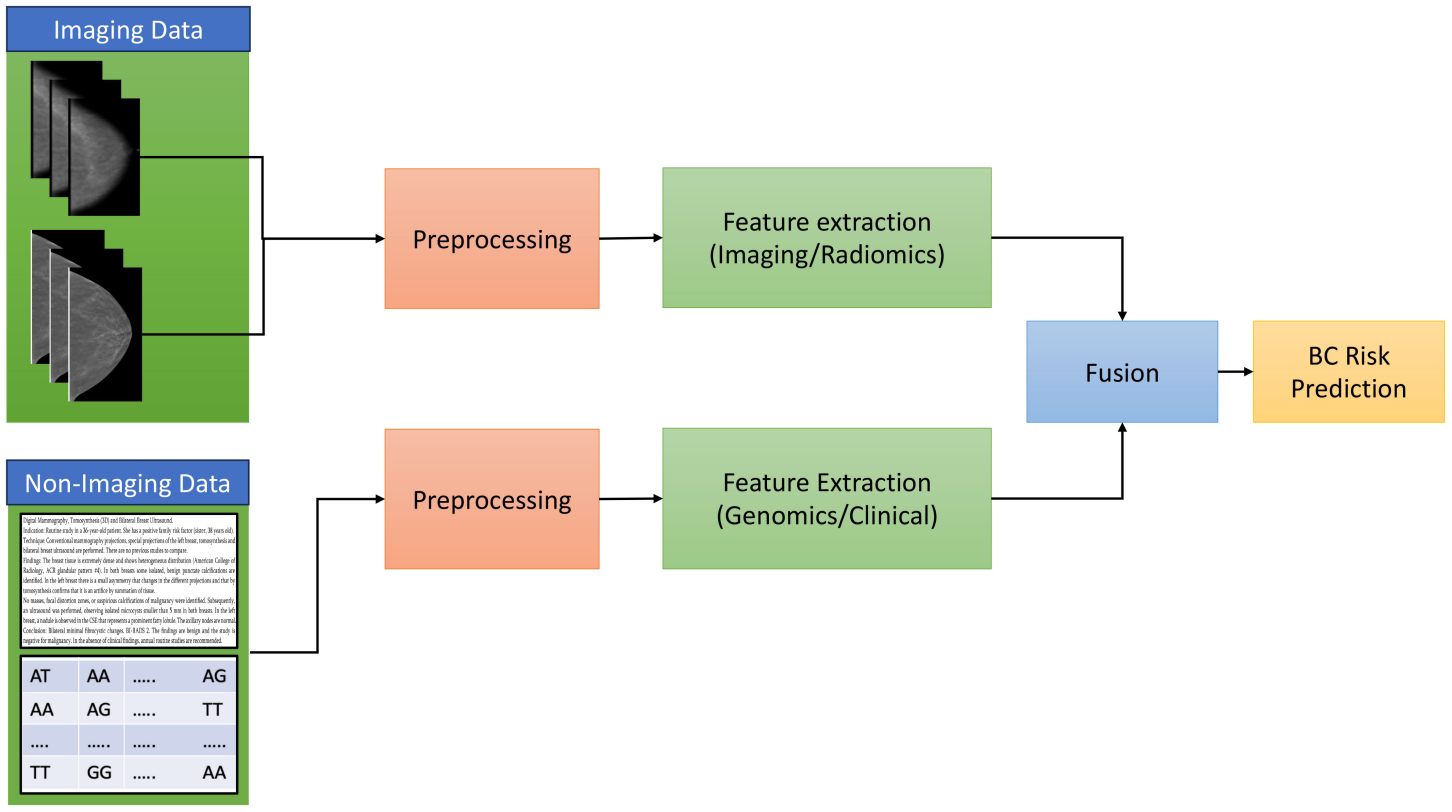
The general overview of breast cancer risk prediction workflow.

**Figure 4:**
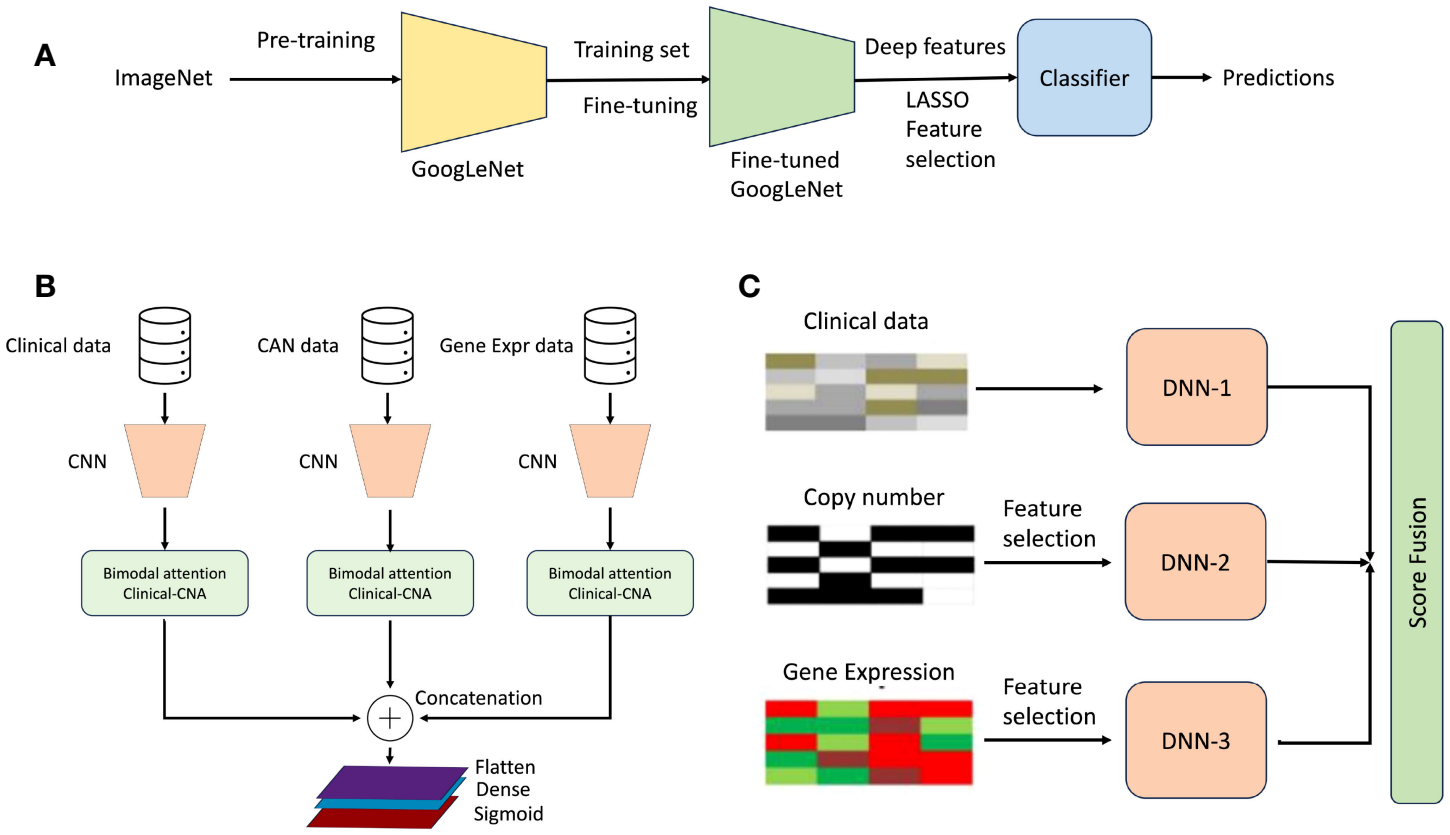
General overview of workflow used in studies on breast cancer risk prediction. **(A)** Proposed model for short-term breast cancer risk prediction. It consists of an end-to-end prediction model using a fine-tuned GoogleNet architecture, which is also used as a deep feature extractor (47). **(B)** General overview of breast cancer risk prediction using multimodal data (48). **(C)** Overall architecture for breast cancer risk prediction. The proposed framework consists of three different models corresponding to each data type and fuses the prediction scores of each model (49).

This section is divided into three subsections: imaging, non-imaging, and imaging and non-imaging studies. In this section, we thoroughly reviewed a body of research dedicated to investigating breast cancer risk prediction by employing state-of-the-art (SOTA) DL techniques. An overview of the risk-prediction workflow is presented in **Figure 3**. A general overview of the architectures used in these studies is shown in **Figure 4**. This comprehensive examination covers diverse studies, each of which provides valuable insights and advances in the area. To facilitate easy reference and access to the details of these studies, we present a summary of their major characteristics and findings in **Tables 1**–**3**. These tables serve as valuable assets for understanding the breadth and depth of investigation of breast cancer risk assessment, highlighting the application of SOTA DL techniques.

**Table 1:** An overview of the ML-based breast cancer risk prediction models using imaging data.

Reference	Dataset Size	Dataset	Model	Result	Prediction
(50)	944	Private	SFFS/SVM	AUC = 0.725	Short-Term
(51)	456	Private	AlexNet/DCNN	AUC = 0.86	Low/High Risk Prediction
(52)	1474	Private	CNN	ACC = 0.72	Low/High Risk Prediction
(53)	133	Private	MLR	AUC = 0.70	Short-Term
(47)	452	Private	GoogleNet-LDA	AUC = 0.73	Short-Term
(54)	2740	OMI-DB	ResNet-18/Transformer	AUC = 0.72	Short-Term

**Table 2:** An overview of the ML-based breast cancer risk prediction models using non-imaging data.

Reference	Dataset Size	Dataset	Model	Result	Prediction
(49)	1980	METABRIC	DNN-SVM/RF/LR	AUC = 0.845	Short/Long Term
(55)	454	Private	SVM-RO-DSS	ACC = 0.86	Short-Term
(56)	3624	Private	AdaBoost	ACC = 0.90	Short/Long Term
(57)	64,739	PLCO	LR	AUC = 0.613	Short-Term
(58)	695	KBCP	XGB	mAP = 77.78	Low/High Risk Prediction
(48)	1980	METABRIC/TCGA-BRCA	GA-CNN	AUC = 0.95	Short/Long Term

**Table 3:** An overview of the ML-based breast cancer risk prediction models using imaging and non-imaging data.

Reference	Dataset Size	Dataset	Model	Result	Prediction
(59)	1,656	Private	ImageNet/ResNet-18	AUC = 0.638	Short-Term
(60)	112,587	Private	AdaBoost	AU-ROC = 0.889	Long-Term
(43)	2,283	Private	Inception-ResNet-v2	AUC = 0.65	Short-Term
(6)	262,318	Private	ResNet-18/Transformer	AUC = 0.76	Short-Term
(61)	5,978	Private	ENLR	AUC = 0.75	Short-Term
(62)	8,604	Private	ProfoundAi Risk	AUC = 0.74	Short/Long Term
(63)	23,467	Private	CNN/LR	AUC = 0.845	Short/Long Term
(64)	171,168	Private	ResNet-18 (ImageNet)	AUC = 0.80	Short/Long Term

#### 3.3.1 Imaging studies

In a study conducted by Tan et al. (50) analyzed breast cancer risk in women with normal mammograms. They utilized mammograms and clinical factors, such as the patient’s age, family history of cancer, and

breast density in a database of 994 women. Among them, 283 patients developed cancer, 349 underwent additional tests, and 362 remained normal. Ten features selected from 183 possibilities were used to train the predictive model. This model, which assigned a score to each case indicating the likelihood of cancer, achieved an AUC score of 0.725 for classifying the positive and negative cases. The model effectively distinguished between cases with and without cancer, indicating its potential to predict breast cancer risk in women with normal mammograms, using features from both breasts.

In a study by Li et al. (51), DL using CNNs was assessed for breast cancer risk prediction. They used full-field digital mammography (FFDMs), including datasets of 106 and 150 FFDMs from high-risk and 328 FFDMs from low-risk patients, respectively. This study compared DL to computerized texture analysis (RTA) to distinguish between high- and low-risk women. DL and RTA performed similarly for BRCA1/2 carriers, but DL outperformed RTA in one-sided breast cancer cases. The fusion classifiers achieved the best AUC of 0.86, suggesting that DL effectively derived features from FFDMs, rivaling or surpassing conventional texture analysis.

Ha et al. (44) developed a new pixel-wise breast cancer risk model using CNNs and mammograms. The study used 420 mammograms from high-risk patients and 1054 from low-risk patients. The CNN-based risk prediction model achieved an AUC of 0.72, proving it to be more effective than breast density in predicting breast cancer risk. This method shows promise for stratifying breast cancer risk without relying on the breast density.

Saha et al. (53) conducted a case–control study involving 133 high-risk women, 46 of whom developed breast cancer within two years, and 87 were used as controls. MRI scans were performed at 3 T or 5 T, using various sequences. A multivariate model based on automated background parenchymal enhancement (BPE) features achieved an AUC of 0.70. These algorithmically extracted imaging features retained their independent predictive value for cancer development, indicating the potential of BPE measurements for improved risk stratification in high-risk women undergoing MRI screening.

Arefan et al. (47) aimed to predict short-term breast cancer risk using DL methods based on normal screening digital mammograms. They used a dataset of 452 mammogram images, including 226 CC and 226 MLO view images. Two DL approaches, GoogLeNet-LDA and end-to-end GoogLeNet, are compared. The GoogLeNet-LDA method achieved the highest AUC of 0.73 for the CC view and 0.72 for the MLO + CC views, demonstrating superior performance in terms of percentage breast density. These findings suggest the effectiveness of DL in improving breast cancer risk assessment based on mammogram images.

In a retrospective study by Damiani et al. (54), a DL algorithm for breast cancer risk prediction using digital mammograms was assessed using the OPTIMAM Mammography Image Database. The study used mammograms from 2,740 patients to achieve an overall AUC of 0.68 for 3-year risk prediction. The model showed consistent performance for interval and screen-detected cancers, was well-calibrated, and was effective in detecting invasive cancer and DCIS. It demonstrated a higher performance in advanced cancer risk prediction, highlighting its potential in early detection strategies.

#### 3.3.2 Non-imaging studies

Sun et al. (49) proposed a new method called Multi-modal Deep Neural Network by integrating Multidimensional Data (MDNNMD) for predicting breast cancer prognosis. They used a dataset of 1,980 valid breast cancer patients from the METABRIC (65) trial, consisting of multimodal data, such as clinical information, gene expression profiles, and CNA profiles. The novel MDNNMD model, which is unique in its design and combination of multidimensional data, achieved an AUC of 0.845. This method outperforms prognosis models using one-dimensional data and other available methods.

Ferroni et al. (55) demonstrated the use of an ML-based decision support system (DSS) and random optimization (RO) to extract predictive insights from routine demographic, clinical, and biochemical data in breast cancer cases. The DSS, trained on 318 patients, demonstrated promise when evaluated on a testing cohort of 136 patients, achieving a C-index of 0.84 for progression-free survival and an accuracy of 86%. It effectively categorized patients into low- and high-risk groups for disease progression, with a hazard ratio (HR) of 10.9 (p ¡ 0.0001). Integrating ML and RO techniques into electronic health record (EHR) data could potentially transform personalized medicine for individual cancer patients.

Ming et al. (56) compared ML models to established breast cancer risk prediction tools (BCRAT and BOADICEA). Using two datasets of samples, 1,143 and 2,481, from US and Swiss breast cancer patients, respectively, the ML-based models showed significantly higher predictive accuracy, reaching 88.28% with ML-Adaptive Boosting and 88.89% with ML-Random Forest for the U.S. population dataset, compared to 62.40% with BCRAT. For the Swiss clinic dataset, ML-Adaptive Boosting achieved 90.17% accuracy, ML-Markov Chain Monte Carlo Generalized Linear Mixed Models 89.32%, while BOADICEA scored 59.31%. These ML algorithms improve the categorization of individuals with and without breast cancer, offering high-precision predictions in personalized medicine.

In a study by Stark et al. (57) ML models were developed to predict five-year breast cancer risk using personal health data. The PLCO dataset (66) consisting of 64,739 women was used. These ML models outperformed the traditional BCRAT based on the Gail model, with the highest AUC of 0.73. Incorporating personal health data can significantly improve the 5-year breast cancer risk prediction accuracy, showing potential as a cost-effective, noninvasive tool for risk assessment.

The study conducted by Behravan et al. (58) aimed to predict breast cancer risk using an ML method that integrates demographic risk factors and interacting genetic variants (SNPs). The KBCP dataset (67), which included demographic risk factors and genotyped data from 250 controls and 445 cases, was used. The approach achieved high prediction accuracy, with tests revealing that combining genetic and Group 1 features resulted in a mean average precision (mAP) of 77.78, surpassing models using only Group 1 features or interacting SNPs. Gene interaction maps provide insights into crucial biological entities related to breast cancer, highlighting the importance of demographic risk factors over genetic variants in predicting breast cancer risk.

An attention-based multimodal DL model was developed (48), which integrates data from copy number alterations and clinical and gene expression sources. A dataset of 1,980 valid breast cancer patients from the METABRIC (65) trial was used. This model utilizes attention mechanisms to analyze mammography images and incorporates patient data to enhance prediction accuracy. The proposed model displays promising results, offering significant potential for improving breast cancer detection and diagnosis.

#### 3.3.3 Imaging and non-imaging studies

Investigators of these findings by Portnoi et al. (59) aimed to establish a DL method using breast MR images to predict the 5-year risk of breast cancer in high-risk women undergoing screening examinations. A dataset of 1,656 MRI examinations from 1,183 unique patients was used for training, whereas 1,623 examinations from 1,164 unique patients were used for model evaluation. The DL model outperformed the conventional risk factor models, achieving an average AUC of 0.638, compared to 0.558 for the risk factor logistic regression (RF-LR) model and 0.493 for the Tyrer-Cuzick (TC) model. The DL model also showed improved individual risk discrimination, suggesting the capability of image-based risk assessment tools for further personalized care.

Ming et al. (60) compared the clinical utility of ML techniques to the BOADICEA model for breast cancer risk prediction and screening. Using data from 112,587 individuals in 2,481 families, ML techniques demonstrated a higher predictive accuracy (0.843 ≤ AU-ROC ≤ 0.889) than BOADICEA (AU-ROC = 0.639) and led to the reclassification of 35.3% of women into different risk categories. The most significant reclassification (20.8%) affected women classified as ‘near population’ risk by the BOADICEA. This reclassification had the most substantial impact on screening practices for women under 50 years of age, emphasizing its importance in clinical decisions regarding screening initiation. This study suggests that the ML-based reclassification of breast cancer risk could significantly influence screening practices, particularly in younger women.

In a study carried out by Dembrower et al. (43), researchers aimed to develop a risk score using a deep neural network (DL) to estimate future breast cancer risk and compared its performance with density-based models. They conducted a retrospective analysis of 2,283 women, 278 of whom were later diagnosed with breast cancer. The DL risk score, derived from digital mammograms, outperformed density-based models. Its accuracy (AUC: 0.65) surpassed the dense area (AUC: 0.60) and percentage density (AUC: 0.57), with a lower false-negative rate (31%) compared to the dense area (36%) and percentage density (39%). This suggests the superior predictive capability of DL over density-based models, particularly for more aggressive cancer cases.

Yala et al. (6) aimed to enhance breast cancer risk models for focused screening using DL. A dataset consisting of 210,819, 25,644, and 25,885 examinations from 56,786, 7,020, and 705 patients, respectively, obtained from the Massachusetts General Hospital (MGH). The Mirai model, developed to predict risk over time and address missing data, was trained on a large dataset, and tested across different populations, achieving high C-indices of 0.76, 0.81, and 0.79 for test sets from various hospitals. Compared to existing models, Mirai significantly outperformed the existing models by providing more accurate 5-year ROC AUCs and better identification of high-risk patients across datasets, including 41.5% on the MGH test set. This demonstrates Mirai’s potential to improve breast cancer risk prediction and the clinical workflow.

A novel short-term breast cancer risk model using digital breast tomosynthesis (DBT) images was developed and validated by Eriksson et al. (61). This model, incorporating imaging features and age, predicts future late-stage and interval breast cancer following negative screening examinations. Involving 805 incident breast cancer cases and 5,173 healthy women who underwent DBT screening, the model displayed strong performance with a discrimination score of 0.82 and effective calibration. According to U.S. Preventive Service Task Force guidelines, 14% of women were identified as high risk, with a 19.6-fold increased risk. In this high-risk group, the model successfully detected 76% of stage II and III cancers and 59% of stage 0 cancers, indicating its potential for earlier detection and improved prognosis. This image-based risk prediction method can aid radiologists in selecting women for clinical care, thereby enhancing breast cancer screening outcomes.

A study conducted by Eriksson et al. (62) compared AI-based short-term breast cancer risk methods using mammograms with traditional lifestyle/familial-based risk methods. Analyzing data from 8,604 women aged 40 to 74 years, the image-based model, which utilized mammographic characteristics and age, outperformed the lifestyle/familial-based model. They achieved higher age-adjusted AUC (aAUC) values for breast cancer prediction over a 10-year period. Impressively, 20% of all women with breast cancer were identified as high-risk using the image-based method compared to 7.1% using the traditional approach. These results indicate the potential value of the image-based risk model for selecting women who may benefit from additional screening and risk-reduction strategies.

Michel et al. (63) assessed the combination of a CNN-based mammographic evaluation with clinical factors for breast cancer risk prediction in 23,467 women undergoing screening mammography. The hybrid model did not substantially enhance risk estimation compared with the clinical factors alone (BCSC model) for the overall cohort (AUC of 0.654 vs. 0.624, p = 0.063). However, the hybrid model in subgroup analyses showed better performance for non-Hispanic Blacks (AUC 0.845 vs. 0.589, p = 0.026) and Hispanics (AUC 0.650 vs. 0.595, p = 0.049). Further validation in a larger cohort is required to explore the potential of the CNN model combined with clinical factors for breast cancer risk assessment in diverse women undergoing screening.

The study carried out by Wang et al. (64) presents the Multi-Time Point Breast Cancer Risk Model (MTP-BCR), an advanced DL risk assessment tool. A large-scale in-house dataset consisting of 171,168 screening mammograms of 9,133 women was used. MTP-BCR utilizes longitudinal mammography data to identify subtle changes in the breast tissue associated with potential future malignancy. With a large dataset of screening mammograms, this model significantly improved long-term (10-year) risk estimation, achieving an AUC of 0.80, outperforming traditional and contemporary methods. It provides unilateral breast-level prognostication, with AUCs of 0.81, 5-year risk and 0.77 for 10-year risk assessments, respectively. The heatmaps of the model aid clinicians in understanding the transition from normal tissue to cancerous progression, enhancing the interpretability of breast cancer risk analysis. This innovative DL approach has the potential to enhance personalized screening and preventive strategies for breast cancer.

## 4 Discussion

The Discussion section of the study emphasizes the ability of AI-based risk assessment models in breast cancer screening, particularly for intermediary and high-risk women who may obtain some advantages from supplementary follow-up. Accurate breast cancer risk analysis plays a crucial role in thorough screening programs, helping identify individuals who require immediate intervention, preventive therapies, and genetic testing, while providing easy, safe, and timely healthcare facilities to the general public.

Traditional clinical algorithms like Gail/BCRAT, BCSC, and Tyrer–Cuzick exhibit varying performances, with AUC values ranging from 0.57 to 0.82 (24). Recent studies have indicated that AI-based models that integrate conventional risk factors with mammographic images can significantly enhance the performance of existing epidemiology-based models. AI techniques applied to datasets from diverse medical centers have reported C-index values in the range of 0.75–0.84 (68), demonstrating promising potential for improved risk assessment.

Different criteria were used to assess model performance, with calibration and discriminatory accuracy being the most common criteria (69). Discriminatory accuracy is often measured using a Concordance Index (C-index). However, it has been observed that only a few studies have employed these metrics, which are appropriate for breast cancer risk assessments.

Given the large number of women receiving annual mammograms in the US, AI offers potential solutions for individualized breast cancer risk assessment and personalized screening schedules. Combining AI models with traditional risk factors can enhance risk analysis, optimize screening strategies, and improve patient outcomes in breast cancer management.

Current breast cancer screening primarily relies on non-imaging risk factors such as family history, demographics, and genetics (24). However, the growing inclusion of mammographic breast density in clinical risk models and the excellence of AI, particularly CNNs, in image analysis, including mammograms, can complement traditional risk factors, such as genetics and hormone status. Recent large-scale studies report promising AI results, with C-index values ranging from 0.75 to 0.84 (68), approaching the performance of established clinical risk models with AUC values between 0.57 and 0.82 (24). Despite this progress, AI-based risk assessments have room for further development.

The 2022 validation investigation by Yala et al. (68) represents a significant study, yet it has limitations. The generalizability of the model can be limited due to homogeneity in patient populations, clinical protocols, and data from a single institute. Additionally, using mammograms from a single vendor may not account for differences in imaging acquisition from other vendors, necessitating the validation of diverse imaging sources. Retraining the model with combined datasets from multiple sources can enhance performance and generalizability.

In 2023, various studies explored AI-based risk analyses for breast cancer. Eriksson et al. (62) compared AI-based risk models with traditional ones, highlighting AI’s potential for long-term risk analysis. Kayikci et al. (48) introduced an attention-based multimodal AI model, while Michel et al. (63) combined CNN-based mammography analysis with clinical factors for promising risk prediction. Damiani et al. (54) evaluated an AI algorithm based on digital mammograms, demonstrating strong predictive capabilities post-negative screenings. Wang et al. (64) introduced the Multi-Time Point Breast Cancer Risk Model, significantly improving long-term risk prediction. These findings emphasize the potential of advanced AI in breast cancer risk assessment and early detection, although further validation in diverse cohorts is vital for its clinical implementation.

There are several limitations in these studies, along with their potential, such as limited data, lack of explainability of methods, and less attention to the ethical aspects of data usage and data privacy considerations.

**Data:** The most common constraints in these studies included the use of homogeneous, unimodal, and single-vendor data. Many studies have relied on data from a single institution. Additionally, these studies often utilized a single modality, such as imaging (mammography or breast MRI), genomics, demographic, or clinical data. It has also been observed that imaging data often originate from a single vendor.
**Short-term risk:** Short-term risk assessments do not reflect the long-term risk of breast cancer. This can limit women’s intention to take preventive measures such as chemoprevention or prophylactic surgery. Short-term risk does not encompass all factors such as genetic or environmental factors (BRCA mutations, lifestyle, and hormonal factors), which can influence breast cancer risk. Women with rare risk profiles require personalized care because complex risk profiles are not adequately assessed by short-term risk prediction models.
**Explainability:** The explainability and interpretability of breast cancer risk prediction methods are among the most under-explored research aspects. Currently, the focus of AI models is on improving accuracy rather than making models explainable. Better explainable models are essential, as they help enhance trust in using AI for breast cancer risk prediction and ultimately improve patient care.
**Ethical Consideration and Data Privacy:** AI-driven breast cancer risk prediction presents both moral and ethical challenges. Previous studies of breast cancer risk prediction studies have focused less on the responsible use of data. It is crucial to ensure that patient data is used responsibly and for the public good. Studies suggest that patients are willing to consent to the use of their data in AI-based breast cancer risk research if they are used confidentially and responsibly with effective governance (70).

Addressing these limitations involves employing multimodal, multi-institutional, heterogeneous data, and providing model explainability. This approach improves the generalizability and performance of models for risk prediction. Moreover, data privacy and ethical considerations should be considered when designing the models, which will ultimately build patient trust in using state-of-the-art AI for breast cancer risk assessment.

## 5 Conclusion

Integrating AI methods, especially DL and CNNs, into breast cancer risk assessment using digital mammography shows great promise for developing personalized risk models. By analyzing individual patient information and medical imaging, these AI-driven approaches have the potential to significantly enhance the accuracy and effectiveness of breast cancer risk prediction. The existing literature demonstrates encouraging results; however, more research and validation are necessary to establish the clinical utility and reliability of these models. As ongoing studies and future applications continue to evolve, the implementation of DL techniques in breast cancer risk modeling could revolutionize screening strategies and facilitate tailored risk management in women worldwide. Given the substantial impact of breast cancer on women’s health, harnessing the power of AI in risk assessment represents a crucial step toward early detection and improved patient outcomes.

## Data availability statement

The original contributions presented in the study are included in the article/supplementary material. Further inquiries can be directed to the corresponding author.

## Author contributions

SH: Conceptualization, Investigation, Methodology, Validation, Writing – original draft, Writing – review & editing. MA: Conceptualization, Visualization, Writing – original draft, Writing – review & editing. UN: Formal analysis, Writing – review & editing. FN: Formal analysis, Writing – review & editing. MJ: Writing – review & editing, Formal analysis. TG: Supervision, Validation, Writing – original draft, Writing – review & editing. JT-P: Conceptualization, Formal analysis, Supervision, Validation, Writing – original draft, Writing – review & editing.
